# Effects of Newer Veterinary Macrolide Antimicrobials on the CYP3A-Dependent Metabolism in Cattle Liver Microsomes: Potential Metabolic Drug–Drug Interaction with Monensin

**DOI:** 10.3390/ani16030378

**Published:** 2026-01-25

**Authors:** Paula Ichinose, Juan Pablo Munafó, María Victoria Miró, Marcela Valente, Laura Moreno-Torrejón, Karen Larsen, Carlos Lanusse, Adrián Lifschitz, Guillermo Virkel

**Affiliations:** 1 Laboratorio de Farmacología, Centro de Investigación Veterinaria de Tandil (CIVETAN), UNCPBA-CICPBA-CONICET, Campus Universitario, Tandil B7000, Buenos Aires, Argentina; paulaichinose@vet.unicen.edu.ar (P.I.); vmiro@vet.unicen.edu.ar (M.V.M.); mvalentemdq@gmail.com (M.V.); lmoreno@vet.unicen.edu.ar (L.M.-T.); kelarsen@vet.unicen.edu.ar (K.L.); clanusse@vet.unicen.edu.ar (C.L.); adrianl@vet.unicen.edu.ar (A.L.); 2 Facultad de Ciencias Veterinarias, Universidad Nacional del Centro de la Provincia de Buenos Aires (UNCPBA), Campus Universitario, Tandil B7000, Buenos Aires, Argentina; 3 Instituto de Investigaciones Bioquímicas de Bahía Blanca (INIBIBB-CONICET), Bahía Blanca B8000, Buenos Aires, Argentina; jpmunafo@inibibb-conicet.gob.ar

**Keywords:** cattle, drug drug interaction, hepatic metabolism, macrolide antimicrobials, monensin

## Abstract

Traditional macrolide antimicrobials are inhibitors of cytochrome P4503A (CYP3A) in cattle liver. Monensin (MON), an ionophore with a narrow safety margin, undergoes CYP3A-dependent O-demethylation, and its incompatibility with macrolides is well recognized in livestock animals. This study evaluated the effects of newer macrolides—tilmicosin (TIL), tulathromycin (TUL), and gamithromycin (GAM)—on CYP3A-dependent metabolism in bovine liver microsomes and examined how these drugs influence MON hepatic metabolism. Molecular docking studies were also performed to predict their interactions with CYP3A enzymes. The CYP3A-dependent enzyme activity, testosterone 6β-hydroxylase, was inhibited in the presence of triacetyl-oleandomycin (used as a reference macrolide), as well as with MON. None of the other macrolides tested affected this enzymatic activity. All macrolides inhibited MON metabolism, but the extent of inhibition observed with triacetyl-oleandomycin was higher than that produced by TIL, TUL, and GAM. Molecular docking analyses indicated that triacetyl-oleandomycin and MON exhibited the highest binding affinities for the active site of CYP3A isozymes, compared with TIL, TUL, and GAM. The agreement between enzymatic data and in silico predictions indicates that TIL, TUL, and GAM are weaker inhibitors of CYP3A-mediated MON metabolism. The modest reduction in MON hepatic metabolism caused by these macrolides—commonly used in cattle feedlots—suggests a low likelihood of clinically relevant drug–drug interactions under typical dosing conditions.

## Simple Summary

The concurrent administration of multiple drugs in cattle feedlot systems can lead to pharmacological drug–drug interactions, particularly those associated with the inhibition of hepatic xenobiotic-metabolizing enzymes. A well-recognized example is the incompatibility between monensin—an ionophore with a narrow safety margin—and traditional macrolide antimicrobials. All these drugs are metabolized by cytochrome P450 (CYP) 3A in bovine liver. We hypothesized that this metabolic interaction could also occur with more recently introduced macrolides, such as tilmicosin, tulathromycin, and gamithromycin. Using an integrated in vitro and in silico approach, the current work shows that these newer macrolides are comparatively weak inhibitors of CYP3A-dependent monensin metabolism. Therefore, the likelihood of a clinically relevant drug–drug interaction under standard dosing conditions is low. Nevertheless, this potential interaction warrants further investigation in vivo, particularly under unintended monensin overexposure.

## 1. Introduction

Feedlots are intensive animal feeding systems where cattle are confined and fed a high-energy diet to promote rapid weight gain before slaughter. These production systems are designed to optimize growth efficiency, enhance meat quality, and maximize output. Since the 1970s, outdoor feedlots have gained worldwide popularity in commercial beef production to shorten the time required for cattle to reach market weight [1].

The concurrent use of multiple drugs is common in cattle confined in feedlot pens. This practice may lead to pharmacological drug–drug interactions (DDIs), particularly those resulting from the inhibition of hepatic xenobiotic-metabolizing enzymes [2]. Such DDIs, occurring as a consequence of enzyme inhibition, can significantly impact drug pharmacokinetics and clinical efficacy. Additionally, they may lead to drug accumulation, increasing the risk of toxicity [2,3]. One of the best-known examples of DDIs is the incompatibility between monensin (MON) and antimicrobial drugs such as tiamulin and certain macrolides [4]. MON, a polyether antimicrobial ionophore, is widely used in cattle feedlots as a growth promoter. It is also indicated for the treatment of coccidiosis and bloat [5]. Macrolide antimicrobials, a class of macrocyclic lactones characterized by a large lactone ring containing 14 to 16 carbon atoms, are among the most frequently administered drugs in feedlot cattle. Within this group, tilmicosin (TIL), tulathromycin (TUL), and gamithromycin (GAM) are primarily used for the prevention and treatment of respiratory diseases, such as pneumonia and bronchopneumonia, mainly caused by *Mannheimia haemolytica*, *Pasteurella multocida*, and *Histophilus somni* [6].

Although MON is considered safe at recommended dosage levels, it possesses a narrow safety margin. Cases of MON toxicity occasionally occur due to misuse or accidental ingestion of excessive ionophore concentrations, often associated with feed mixing errors [7,8,9]. In cattle and other food-producing species, the ionophore undergoes O-demethylation catalyzed by cytochrome P450 isozymes (CYPs) of the CYP3A subfamily [10]. Tiamulin and certain macrolide antimicrobials have been shown to form stable metabolic intermediate (MI) complexes with the active site of CYP3A isozymes [11,12]. These MI complexes, formed during drug metabolism, inhibit CYP3A activity and reduce the biotransformation rate of simultaneously administered drugs. Consequently, macrolide antimicrobials may impair MON metabolism, leading to its accumulation and an increased risk of toxicity. In fact, adverse toxicological effects have been reported in feedlot cattle following the consumption of MON-containing feed contaminated with macrolide antibiotic residues [13].

The formation of MI complexes and the inhibition of CYP3A-dependent metabolism by macrolides and tiamulin have been demonstrated in vitro using cattle liver microsomes and a cell line overexpressing a bovine CYP3A isozyme [11]. These authors reported that tiamulin and triacetyloleandomycin (TAO) are potent CYP3A inhibitors, while erythromycin and TIL act as relatively weaker inhibitors. We hypothesize that similar metabolic interactions could occur with more recently introduced macrolides, such as TUL and GAM. Therefore, the present work aimed to assess the effects of TAO, TIL, TUL, GAM, and MON on CYP3A-dependent metabolism in cattle liver microsomes, and to evaluate the impact of the macrolide antimicrobials on the hepatic metabolism of MON. Additionally, in silico molecular docking studies were conducted to predict DDIs among these compounds with bovine CYP3A enzymes.

## 2. Materials and Methods

### 2.1. Chemicals

Reference standards of MON, TAO, TIL, TUL, and GAM were obtained from Merck (Buenos Aires, Argentina). Testosterone (4-androsten-17β-ol-3-one) and its 6β- (4-androsten-6β, 17β-diol-3-one) hydroxylated metabolite were from Steraloids (Newport, RI, USA). HPLC solvents used for sample dilution and chromatographic analysis were purchased from J.T Baker-Avantor (Phillipsburg, NJ, USA). Sterile water was obtained from Tecsolpar SA (Balcarce, Argentina). Buffer salts (KCl, NaHCO_3_, Na_2_HPO_4_, NaH_2_PO_4_, K_2_HPO_4_, KH_2_PO_4_, and CH_3_COONH_4_) were purchased from J.T Baker-Avantor (Phillipsburg, NJ, USA), whilst all other chemicals were from Merck (Buenos Aires, Argentina).

### 2.2. Animals and Preparation of Liver Microsomes

Cattle liver samples were obtained from four (4) Aberdeen Angus/Hereford crossbreed steers, weighing approximately 350 kg (18–20 months old), at a local slaughterhouse located 16 km away from the laboratory facilities (Mirasur SA, Tandil, Argentina). Animals were rendered unconscious by a captive bolt and immediately exsanguinated. These procedures agreed with the Animal Welfare Policy (Academic Council Resolution 087/02) of the Faculty of Veterinary Sciences, Universidad Nacional del Centro de la Provincia de Buenos Aires (UNCPBA), Tandil, Argentina (Internal Protocol 16/2020; approval date: 27 November 2020).

After slaughter, the whole liver was collected directly from the animal carcass and placed on a tray. A sample of approximately 10 g from the caudate lobe was excised with a scalpel, rinsed with ice-cold 1.15% KCl to remove hemoglobin, covered with aluminum foil, snap-frozen immediately in liquid nitrogen, and stored at −80 °C until use. Microsomal fractions from hepatic tissue were isolated by differential ultracentrifugation as previously described [14]. An aliquot of each subcellular fraction was used to determine protein content according to Lowry et al. (1951) [15].

### 2.3. Measurement of CYP Content

CYP content was determined using a PG Instruments T80+ UV-Vis spectrophotometer (PG Instruments Limited, Wibtoft, Leicestershire, UK) by recording the carbon monoxide difference spectrum (450–490 nm) of sodium dithionite–reduced microsomal suspensions, applying an extinction coefficient of 104 mM^−1^·cm^−1^ [16].

### 2.4. Demethylase Activities

The O-demethylation of MON and the N-demethylation of various macrolide antimicrobials (TAO, TIL, TUL, and GAM) were assessed using previously reported methodologies [10,17]. All drugs were dissolved in 5 µL of ethanol to achieve a final concentration of 300 µM in a 1 mL incubation assay. Each drug was independently incubated in a NADPH-regenerating system containing NADP+ (0.32 mM), glucose-6-phosphate (6.4 mM), MgCl_2_ (5 mM), EDTA (0.8 mM), and glucose-6-phosphate dehydrogenase (1.25 U) in Tris-HCl buffer (0.1 M, pH 7.4), along with 1 mg of microsomal protein. After a 15-min incubation at 37 °C in an oscillating water bath, reactions were quenched with chilled trichloroacetic acid (10% *w*/*v*). Following centrifugation, the released formaldehyde was quantified fluorometrically from the clear supernatant using Nash’s reagent, as described by Werringloer (1978) [18], with a Varioskan™ LUX Multimode Microplate Reader (Thermo Fisher Scientific, Waltham, MA, USA).

### 2.5. Testosterone Hydroxylase Activity

Testosterone 6β-hydroxylation was assessed as a marker of CYP3A-dependent metabolism in the absence and presence of macrolides, MON, and their combinations, following the method described by Zweers-Zeilmaker et al. (1999) [11] with slight modifications. Briefly, metabolic assays were conducted individually using 0.5 mg of microsomal protein dissolved in the NADPH regenerating system, with a total volume of 0.5 mL. Microsomes were preincubated (30 min, 37 °C) with either 25 or 125 µM of the tested macrolides or 250 µM of MON. The macrolide concentrations were selected based on their ability to promote MI complex formation (125 µM) or significantly inhibit CYP3A activity (25 and 125 µM) in liver microsomes from food-producing species [11,12], while the MON concentration was previously employed for evaluating its hepatic metabolism [10]. All drugs were dissolved in 5 µL of methanol. Testosterone (10 µL of a 10 mM solution in methanol) was then added to reach a final concentration of 200 µM. Control assays (without macrolides or MON) contained the same additional methanol volume. For each microsomal preparation, two independent technical replicates were performed under each experimental condition. All incubations were conducted under aerobic conditions for 15 min at 37 °C in an oscillating water bath, after which they were quenched with 400 µL of ice-cold acetonitrile. HPLC analysis was performed as described by Pegolo et al. (2010) [19] using a Shimadzu 10 A HPLC system (Shimadzu Corporation, Kyoto, Japan).

### 2.6. MON Metabolism

The total microsomal metabolism of MON was assessed by the rate of disappearance of the ionophore either in the absence (control incubations) or in the presence of the various macrolides. Metabolic assays were conducted individually using 0.5 mg of microsomal protein dissolved in the NADPH regenerating system, with a total volume of 0.5 mL. Microsomes were preincubated (30 min, 37 °C) with 15 µM of the tested macrolides. All drugs were dissolved in 5 µL of methanol. MON (10 µL of a 10 mM solution in ethanol) was then added to reach a final concentration of 0.75 µM. Control assays (without macrolides) contained the same additional methanol volume. For each microsomal preparation, two independent technical replicates were performed under each experimental condition. All incubations were carried out under aerobic conditions for 30 min at 37 °C and were quenched with 500 µL of ice-cold acetonitrile. The MON concentration was within the range observed in the liver of cattle fed a diet containing 30 g of the ionophore per U.S. ton [20]. The macrolide concentration was estimated using reported ranges of tissue penetration coefficients for each drug [21,22,23].

MON concentrations in microsomal incubation samples were determined using an Ultra Fast Liquid Chromatography–Tandem Mass Spectrometry (UFLC–MS/MS) system from Shimadzu (Shimadzu Corporation, Kyoto, Japan). The UFLC system consisted of two Shimadzu LC-20AD Prominence pumps, a Shimadzu SIL-20AC HT Prominence injector, and a Shimadzu CTO-20AC Prominence column oven. Mass detection was performed on a Shimadzu LCMS-8050 triple quadrupole mass spectrometer. A Kromasil Eternity C18 analytical column (100 mm length × 2.1 mm internal diameter, 2.5 µm particle size; Sigma-Aldrich, Nouryon, Sweden) at 40 °C (oven) was used. The mobile phase consisted of acetonitrile and 5 mM ammonium formate, using a gradient elution as follows: 60:40 (initial conditions), 95:5 (2.5–9 min), and 60:40 (9.5–13 min), at a flow rate of 0.2 mL/min. To precipitate microsomal proteins, inactivated MON incubations were centrifuged at 13,800× *g* for 20 min at 4 °C. Aliquots of the resulting supernatants were then diluted 1:10 with the initial mobile phase, and a 2 μL volume was injected into the UFLC–MS/MS system for analysis.

The mass spectrometer was operated in positive mode electrospray ionization (ESI) and in multiple reaction monitoring (MRM) mode. The most abundant precursor ions and their corresponding fragment ion transitions for MON were monitored. The dwell time was set to 100 ms for both the quantifier and qualifier ions. The temperatures of the interface and desolvation line were maintained at 250 °C, while the heating block was set to 400 °C. The gas flows were 8 L/min for the heating gas (air), 3 L/min for the nebulizer gas (N2), and 8 L/min for the drying gas (N2). The collision gas (argon) pressure was kept at 270 mPa. Optimization of collision energies (in eV) was performed through direct injections of 0.1 mg/mL MON standard in the mass spectrometer. The retention time, collision energy, and the quantifier and qualifier ions for MON are presented in Table 1.

A validation of the analytical procedures for MON quantification was conducted before analyzing the experimental samples from the incubation assays. Briefly, known amounts of the analyte were added to aliquots of inactive microsomes diluted in the incubation buffer to obtain calibration standards (7–75 nM), which were analyzed by UFLC MS/MS in triplicate. A calibration curve was generated using least squares linear regression analysis (Instat 3.00, Graph Pad Software, Inc., San Diego, CA, USA), correlating UFLC peak areas with the nominal concentrations of spiked samples. The coefficient of determination (R^2^) was 0.999. A lack-of-fit test was also performed to confirm the linearity of the regression line for MON. The concentrations in the experimental samples were determined by interpolating the peak areas of the ionophore measured in the experimental samples against the standard curve. Accuracy was assessed following the HPLC MS/MS analysis of replicates of two concentrations of MON (14 and 36 nM) prepared in inactive (diluted) microsomes, with relative errors (RE%) remaining below 15%.

### 2.7. In Silico Studies

A comparative analysis of sequence identity and structural conservation was conducted between human CYP3A4 and bovine CYP3A isozymes. The amino acid sequences of human CYP3A4 (CYP3A4; UniProt accession: P08684), and bovine CYP3A28 (CYP3A28; UniProt accession: Q29RV2; ENSBTAG00000049666), CYP3A74 (CYP3A74; UniProt accession: A0A3Q1LK48; ENSBTAG00000052665), and CYP3A76 (CYP3A5 Bos taurus; UniProt accession: Q3T047; ENSBTAG00000053645) were retrieved from the UniProt database (https://www.uniprot.org/; accessed on 18 March 2025) [24]. AlphaFold-predicted three-dimensional structures for proteins were downloaded in PDB format. The primary sequence of human CYP3A4 was individually aligned with that of bovine CYP3A28, CYP3A74, and CYP3A76 using the pairwise alignment tool ALIGN (https://www.uniprot.org/align; accessed on 18 March 2025) [24]. To quantify sequence conservation between human CYP3A4 and the bovine isoforms, the percentages of identity and similarity were calculated using the “Identify Similarity” tool from the Sequence Manipulation Suite (https://www.bioinformatics.org; accessed on 18 March 2025) [25]. Structural superposition of the AlphaFold models was performed using UCSF ChimeraX version 1.9 [26]. The structures were aligned based on their backbone atoms, and root-mean-square deviation (RMSD) values were calculated to assess structural divergence. Visualizations of aligned structures were generated to highlight conserved and divergent regions.

Alignment of the complete structures corresponding to human CYP3A4 and each of the bovine isoforms—CYP3A28, CYP3A74, and CYP3A76—and visualization of the molecular docking results were performed using UCSF ChimeraX. This software, developed by the Resource for Biocomputing, Visualization, and Informatics at the University of California, San Francisco, is supported by the National Institutes of Health (R01-GM129325) and the Office of Cyber Infrastructure and Computational Biology, National Institute of Allergy and Infectious Diseases (NIAID) [26].

To further investigate the interactions between the antimicrobial compounds and CYP3A4, molecular docking studies were performed using the crystal structure of human CYP3A4 co-crystallized with ritonavir (RIT), which represents an experimentally resolved inhibited conformation (PDB ID: 5VC0) [27]. Antimicrobials were designed using Avogadro 1.2.0 and prepared for docking with UCSF Chimera 1.16 [28]. Docking simulations were carried out using AutoDock Vina 1.1.2 [29,30,31] via the Chimera interface. Before docking, the protein-ligand systems were prepared by removing water molecules and adding hydrogen atoms at pH 7.4. Gasteiger charges were assigned, and default parameters were maintained for the docking simulations. The grid was defined around the orthosteric binding site of CYP3A4. One hundred genetic algorithm runs were performed for each condition. The docking results were corroborated through six distinct procedures. Protein-ligand interactions were analyzed using Chimera’s “Find Clashes/Contacts” tool and the Protein-Ligand Interaction Profiler (PLIP) [32].

### 2.8. Data Handling and Statistical Analysis

Demethylation rates of MON and macrolide antimicrobials were expressed as nmol of formaldehyde released per min per mg of microsomal protein (nmol/min.mg) and per nmol of CYP (nmol/min.nmol CYP). Testosterone 6β-hydroxylase activity was expressed as nmol of the metabolite formed per min per nmol of CYP. The rate of disappearance of MON from the microsomal incubation medium was expressed as pmol per min per mg of microsomal protein. All data were expressed as the arithmetic mean ± standard deviation (SD). Data normality (Gaussian distribution) was evaluated using the Shapiro–Wilk test in GraphPad Prism 8.1.0 (GraphPad Software, Boston, MA, USA). Statistical comparisons were performed using the same software. Demethylase activities were statistically compared by means of ANOVA for paired observations followed by Tukey’s multiple comparison test. All other metabolic activities were compared by means of Brown-Forsythe and Welch ANOVA tests followed by Games–Howell’s multiple comparison test. In all cases, differences were considered statistically significant when *p* values were below 0.05.

## 3. Results

The CYP content in liver microsomes from steers was 0.32 ± 0.08 nmol/mg of microsomal protein. Table 2 shows the comparative O-demethylation of MON and N-demethylation of the four macrolide antimicrobials in cattle liver microsomes. No statistically significant differences were found among their demethylation rates.

**Figure 1:**
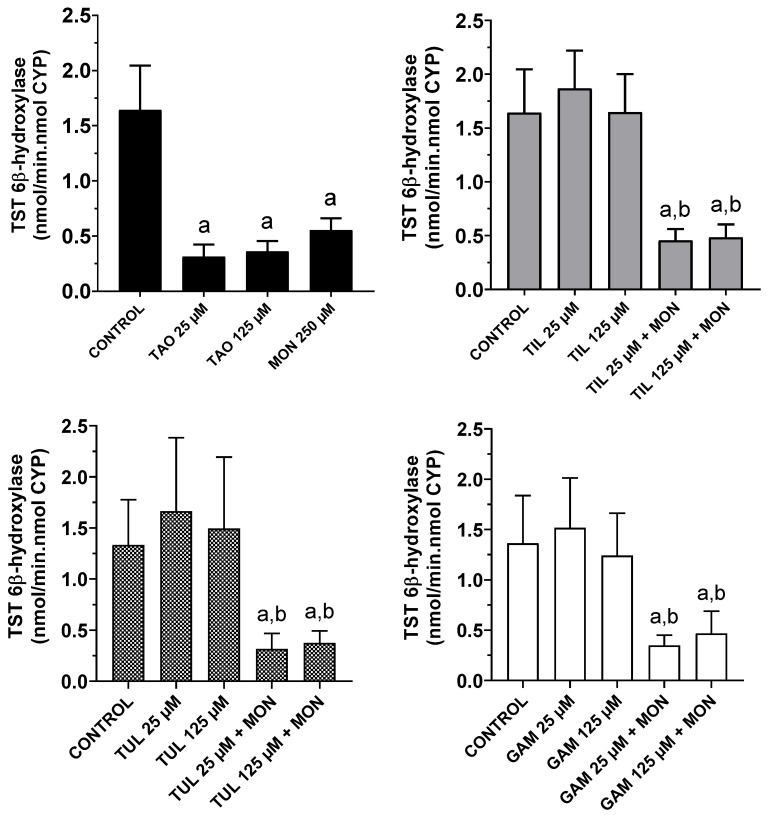
Effects of monensin (MON), triacetyloleandomycin (TAO), tilmicosin (TIL), tulathromycin (TUL), gamithromycin (GAM), and the combinations of MON with three macrolides (TIL, TUL, and GAM) on testosterone (TST) 6β-hydroxylase activity in cattle liver microsomes. Data are the mean (±SD) of eight determinations. a: Significantly different (*p* < 0.05) vs. control incubations. b: Significantly different (*p* < 0.05) vs. incubations performed with the macrolide antimicrobial alone.

Figure 1 illustrates the effects of MON, TAO, TIL, TUL, GAM, and the combinations of MON with three macrolides (TIL, TUL, and GAM) on testosterone 6β-hydroxylation in cattle liver microsomes. Significant inhibition (*p* < 0.05) of this enzyme reaction was observed in the presence of TAO at both 25 µM (81%) and 125 µM (78%), as well as with MON alone (66%). Increasing the TAO concentration fivefold did not result in a statistically significant change in 6β-hydroxytestosterone production. None of the other macrolides tested at 25 or 125 µM significantly affected this enzymatic activity. Furthermore, their respective combinations with MON elicited inhibition rates (66–76%, *p* < 0.05) comparable to that observed with MON alone.

**Figure 2:**
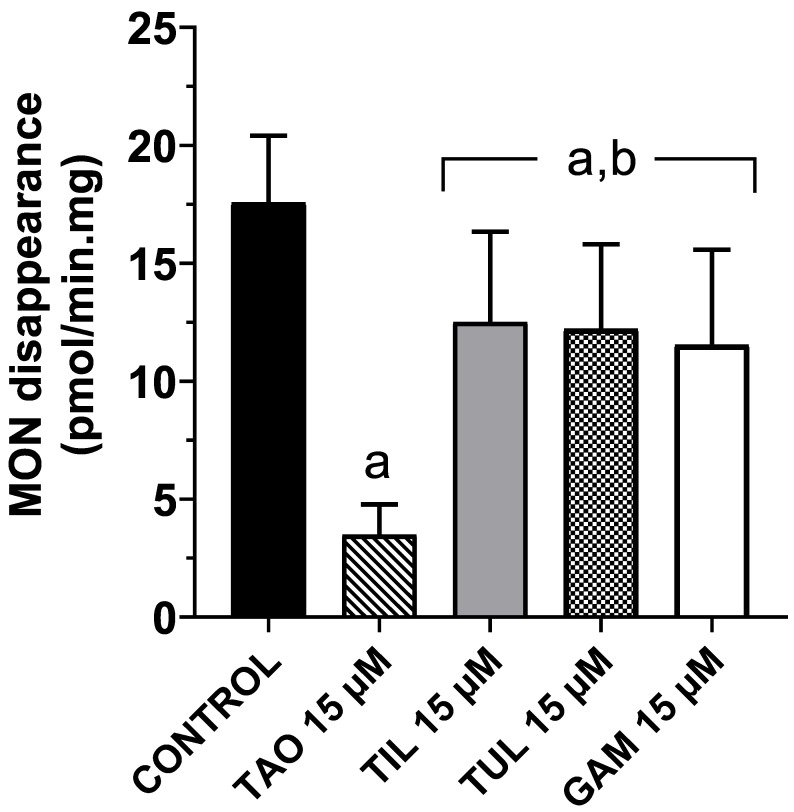
Effects of different macrolide antimicrobials on the metabolism of the ionophore monensin (MON) in cattle liver microsomes. TAO: triacetyloleandomycin; TIL: tilmicosin; TUL: tulathromycin; GAM: gamithromycin. Data are the mean (±SD) of eight determinations. a: Significantly different (*p* < 0.05) vs. control incubations (MON alone). b: Significantly different (*p* < 0.05) vs. MON incubations in the presence of TAO.

Figure 2 illustrates the effects of four macrolide antimicrobials on the hepatic metabolism of MON in cattle, assessed by the measurement of the ionophore’s disappearance from the incubation medium of liver microsomes. All macrolides significantly inhibited (*p* < 0.05) MON metabolism. However, the extent of inhibition observed with TAO (80%) was significantly greater (*p* < 0.05) than that produced by TIL (28%), TUL (30%), and GAM (34%).

**Figure 3:**
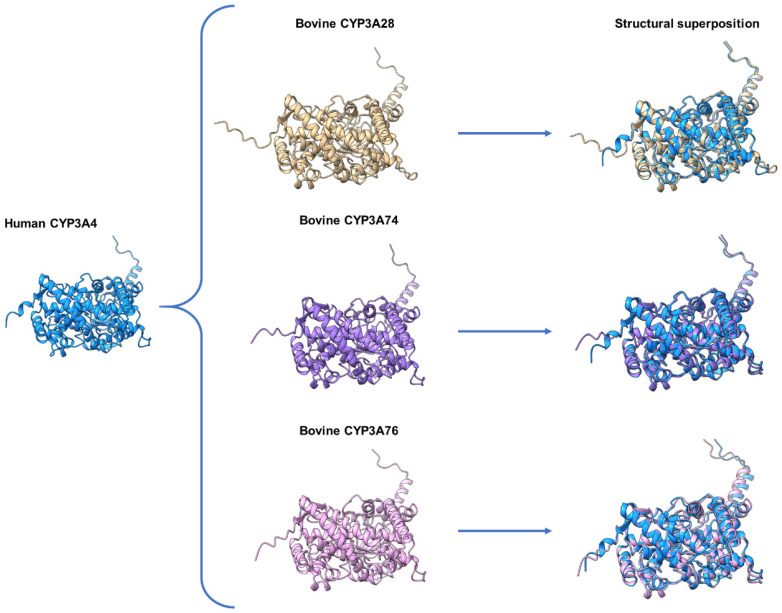
Structural comparison between human cytochrome P450 (CYP) 3A4 and its bovine orthologs. Three-dimensional structures of the human isoform CYP3A4 (cyan, left) were compared with three bovine orthologs: CYP3A28 (beige), CYP3A74 (purple), and CYP3A76 (pink). The central panels show the individual structures of each bovine isoform. The panels on the right displayed the structural superpositions of each bovine isozyme with human CYP3A4.

Figure 3 shows the structural comparison between human CYP3A4 and the bovine isoforms CYP3A28, CYP3A74, and CYP3A76. Alignment of human CYP3A4 with bovine CYP3A28 yielded 507 aligned residues, with 362 identical and 41 similar residues, corresponding to 71.4% identity and 79.5% similarity. Structural superposition produced an RMSD of 0.595 Å across 483 pruned atom pairs (1.099 Å across all 498 pairs). In comparison, alignment with bovine CYP3A74 included 503 residues, with 388 identical and 44 similar residues—equating to 77.1% identity and 85.9% similarity. Structural alignment resulted in an RMSD of 0.587 Å over 492 pruned atom pairs (0.907 Å across all 503 pairs). Similarly, alignment with CYP3A76 yielded 503 aligned residues, including 388 identical and 41 similar residues (77.1% identity and 85.3% similarity), and an RMSD of 0.570 Å over 472 pruned atom pairs (1.190 Å across all 503 pairs).

**Figure 4:**
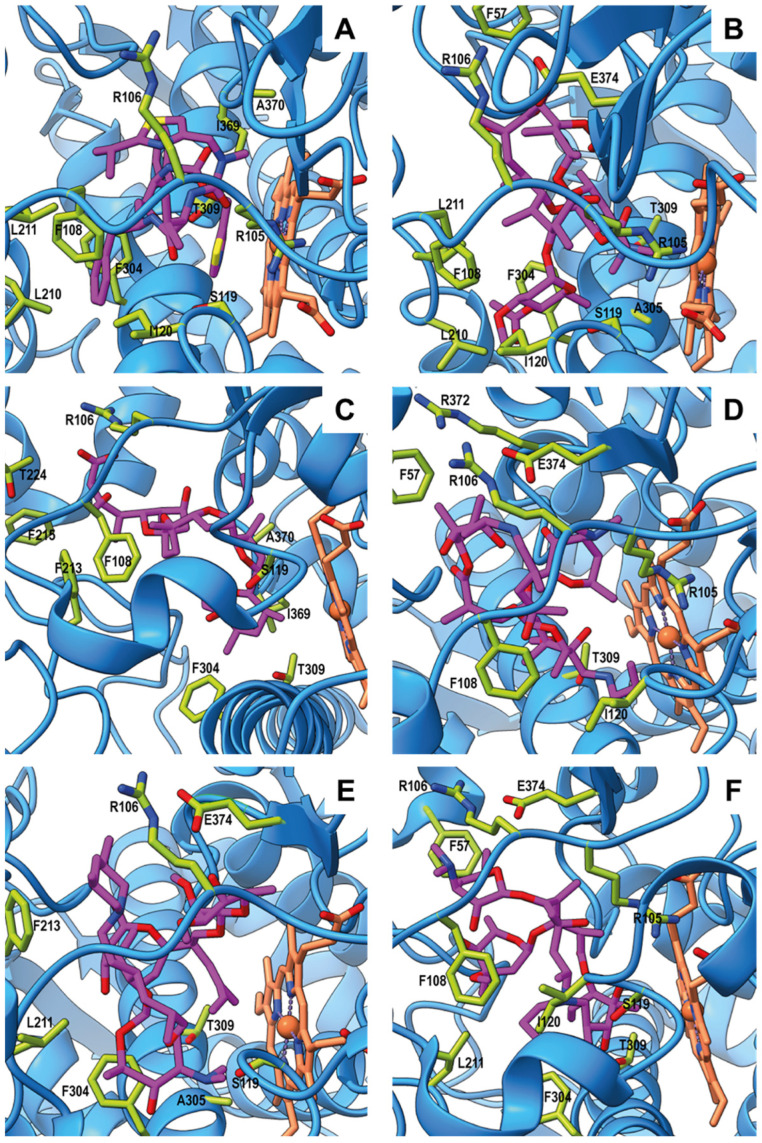
Molecular docking into the three-dimensional structure of the active site of human cytochrome P450 3A4 showing the best binding conformation for: (**A**) ritonavir, (**B**) triacetyloleandomycin, (**C**) monensin, (**D**) tulathromycin, (**E**) tilmicosin, and (**F**) gamithromycin. Ligands are shown as violet sticks; surrounding residues in the binding pocket as green sticks; the heme group with its iron atom as orange; and the enzyme backbone as light blue.

Molecular docking simulations were performed using a CYP3A structure in its inhibited conformation, crystallized in complex with RIT (PDB ID: 5VC0). Re-docking of the co-crystallized RIT was first performed to define the binding site, ensuring that the location and geometry of the binding cavity were consistent with the ligand-bound crystal structure. Following this, RIT was removed, and the tested compounds (macrolides and MON) were evaluated within the defined binding cavity. Table 3 lists the interacting amino acid residues identified in these docking analyses, and the best binding energy (BBE) corresponding to the most stable conformation identified through docking analysis. Figure 4 illustrates the three-dimensional structure of the human CYP3A4 active site, showing the best binding conformation for the drugs tested in silico.

## 4. Discussion

Macrolide antimicrobials are known to undergo CYP3A-dependent metabolism in humans [33] and in veterinary species [17]. The main metabolic pathway for this class of drugs is N-demethylation, and the rate of this reaction is widely accepted as a marker of CYP3A enzymatic activity in cattle liver [17,34,35,36]. Similarly, MON undergoes CYP3A-mediated O-demethylation in food-producing animals [10]. The metabolic rates determined in this study for TAO N-demethylation and MON O-demethylation (see Table 2) were of the same order of magnitude as those previously reported in cattle liver microsomes [10,17,36]. While the O-demethylation of MON and the N-demethylation of 14-membered macrolides, such as TAO and erythromycin, are well characterized [10,17,34,35,36,37], information regarding the metabolism of 15- and 16-membered macrolides (e.g., TIL, TUL, and GAM) remains limited. In this regard, residue-depletion, pharmacokinetic and biotransformation studies have demonstrated that TIL undergoes hepatic N-demethylation in cattle [38,39] and in rabbits [12]. The N-demethylation rate of TIL observed in the present study was consistent with that previously reported in rabbit liver microsomes. Conversely, TUL was found to be minimally metabolized, with biliary excretion of the parent compound being the predominant elimination route, and N-demethylated metabolites accounting for less than 10% of the total residues [40]. For GAM, available pharmacokinetic data indicate that the parent molecule is mainly excreted unchanged, suggesting that biotransformation plays a minor role in its overall disposition [21,41]. In the current work, we evaluated the comparative N-demethylation of these macrolides, along with the O-demethylation of MON (see Table 2). Because all drugs were incubated at the same concentration, the absence of significant differences in their metabolic rates may indicate a comparable affinity for the CYP3A catalytic site, suggesting that none of the compounds acted as a preferential substrate under the tested conditions.

A strong inhibition of CYP3A-dependent testosterone 6β-hydroxylase activity was observed in the presence of TAO at both 25 and 125 µM (see Figure 1), consistent with previous results obtained using cattle liver microsomes [11]. Although all macrolide antimicrobials share a similar core chemical structure, some members of this class are weaker inhibitors because they form only weak metabolic-intermediate (MI) complexes with the enzyme’s active site. For instance, compared with TAO, erythromycin and TIL have been reported to weakly inhibit testosterone 6β-hydroxylase activity in cattle liver microsomes [11]. Accordingly, in the present study, no inhibition of this CYP3A-mediated enzymatic reaction was detected in the presence of TIL, TUL, or GAM. This finding suggests a limited ability to form MI complexes with CYP3A enzymes and is consistent, at least for TIL, with previous results obtained using liver microsomes from rabbits pretreated with the CYP3A inducer rifampicin [12].

The available information on the effects of ionophores on hepatic CYP-dependent metabolism is limited. In this regard, the present study demonstrated that MON, when incubated at 250 µM, inhibited testosterone 6β-hydroxylase activity in cattle liver microsomes (see Figure 1). Clearly, both MON and testosterone may compete for the active site of CYP3A isozymes expressed in bovine liver. However, the present study did not investigate the mechanistic basis of this substrate competition. Although most biotransformation studies indicate that MON behaves primarily as a substrate rather than a CYP inhibitor, experimental data suggest that it can modify microsomal NADPH oxidation rates and produce type I binding spectra, consistent with interaction at the heme-thiolate site of CYP enzymes [42]. However, no appreciable changes in total CYP content or inhibition of several oxidative CYP-dependent activities, NADPH–cytochrome c reductase, or glutathione S-transferase were observed in liver microsomes from untreated or phenobarbital-treated rats incubated with the ionophore at the same concentration (250 µM) [42]. Nevertheless, that work did not assess a CYP3A-mediated metabolic reaction such as the one examined in the present study.

All macrolides tested inhibited the metabolism of MON in cattle liver microsomes (see Figure 2). A marked inhibition of MON disappearance from the incubation medium was observed in the presence of TAO, consistent with previous reports describing the inhibitory effects of this macrolide and tiamulin on the ionophore’s metabolism in rat liver microsomes [37,43]. In contrast, only a slight reduction in MON metabolism was observed in the presence of TIL, TUL, and GAM. This finding agrees with their lack of inhibition of the CYP3A-dependent testosterone 6β-hydroxylase activity. Consequently, the potential interference of these macrolides with the hepatic metabolism of MON appears to be of limited clinical relevance. Nevertheless, an increased risk of this DDI could arise under conditions of unintended subclinical overexposure to the ionophore. In this regard, Puschner et al. (2016) [44] demonstrated that increasing the oral MON dose from 1 mg/kg (the recommended level) to 5 mg/kg for 10 consecutive days resulted in approximately fivefold higher ionophore concentrations in the liver, kidney, and heart of dairy cows. The high-dose group (5 mg/kg) exhibited clinical signs including diarrhea, reduced feed intake, and depression, along with elevated serum cardiac troponin I levels, indicative of cardiomyocyte injury following repeated exposure. Based on these observations, the potential contribution of macrolide antimicrobial treatments to MON toxicosis, particularly under conditions of overexposure, warrants further investigation.

Molecular docking studies were conducted to characterize the interactions between the antimicrobial compounds and CYP3A isozymes. Sequence and structural analyses revealed a high degree of homology between human CYP3A4 and the bovine isoforms CYP3A28, CYP3A74, and CYP3A76 (see Figure 3). The identity and similarity values obtained between human CYP3A4 and the bovine CYP3A isozymes were consistent with those previously reported [45,46]. To explore the molecular interactions between CYP3A4 and the macrolides or MON, docking simulations were performed using the crystal structure of human CYP3A4 complexed with RIT [27]. This drug acts as a potent inhibitor of CYP3A4 by binding directly to its active site [47]. The inhibitory mechanism is mediated by specific interactions between RIT and key active-site residues, particularly S119, involving hydrogen bonding, hydrophobic contacts, and coordination with the heme group. These interactions stabilize RIT within the active site and induce a rigid, “closed” conformation of the enzyme, thereby preventing substrate access and resulting in competitive inhibition [27].

For the present molecular docking analyses, the grid dimensions were restricted to the region encompassing the active site, and six independent docking runs were performed for each compound. As an internal control, re-docking simulations were first conducted with RIT. The docking results should be interpreted within the context of the structural model used, which corresponds to a human CYP3A crystal structure co-crystallized with RIT and represents an experimentally resolved inhibited conformation. This structure was selected not only because it reflects an inhibitory binding state but also due to its high sequence and structural homology with bovine CYP3A isozymes (see Figure 3), supporting its suitability as a model for exploring ligand interactions at the conserved active site. To the best of our knowledge, no experimentally resolved structure of bovine CYP3A in this inhibited conformation is currently available. Within this structural framework, RIT serves as a reference ligand that defines the location and geometry of the binding cavity in the selected model. Under these conditions, the most stable conformation yielded a binding energy of approximately −10.5 kcal/mol. RIT formed hydrogen bonds with residues S119 and I369 and π-stacking interactions with F304. In addition, hydrophobic contacts were observed with R105, R106, F108, L210, L211, I120, F304, T309, and A370. Several of these interactions (see Table 3) correspond to those reported in the crystal structure [27] (see Figure 4).

Molecular docking allowed the analysis of the interactions of each compound (macrolides and MON) with CYP3A within a fixed protein conformation associated with inhibition. All compounds tested in the current work interacted with key residues within the active site of CYP3A4, including S119, F108, I120, L210, L211, F304, A305, T309, I369, and A370. These residues are known to be critical for substrate and inhibitor binding in this isozyme, as demonstrated by site-directed mutagenesis and homology modeling studies [48]. The ionophore MON and all macrolides were positioned within the active site, establishing interactions with residues implicated in enzyme inhibition. MON exhibited a binding affinity of −10.6 kcal/mol, forming hydrogen bonds with R106, S119, and T309, a salt bridge with R106, and hydrophobic interactions with F108, F213, F215, T224, F304, E308, T309, I369, and A370. These findings are complementary to recent in silico studies addressing species-related differences in MON toxicity, which provided the first structural evidence underscoring the pivotal role of CYP3A-mediated O-demethylation in the detoxification of the ionophore [49].

The predominant docking conformation of TAO—a well-known CYP3A inhibitor used in experimental activity assays—yielded a binding score of approximately −10.9 kcal/mol, the highest among all tested compounds. This ligand formed hydrogen bonds with R106, S119, T309, and E374, along with hydrophobic contacts involving F57, R105, F108, I120, L210, L211, F304, and A305. Newer macrolides (TUL, TIL and GAM) showed comparatively lower binding scores relative to both TAO and MON. TUL (−10.2 kcal/mol) engaged in hydrogen bonds with T309, R372, and E374, and hydrophobic contacts with F57, R106, F108, and I120. Notably, it was the only macrolide that did not form a hydrogen bond with S119. TIL (−10.0 kcal/mol) formed hydrogen bonds with R106, S119, and E374, accompanied by hydrophobic interactions with L211, F213, F304, and A305. GAM (−9.9 kcal/mol) established hydrogen bonds with S119 and T309, a salt bridge with E374, and hydrophobic contacts with F57, R105, R106, F108, I120, L211, and F304. Overall, these findings indicate that TAO and MON exhibit a greater binding affinity for CYP3A4 than TUL, TIL, and GAM. The in vitro data closely mirrored the computational results, confirming that TAO and MON produced the strongest inhibition of CYP3A-dependent activity. This agreement between molecular docking and enzymatic assays supports the reliability of the computational approach for predicting inhibitory potential. Likewise, the lower binding scores obtained for TIL, TUL, and GAM, relative to TAO, are consistent with their weaker inhibitory effects on MON metabolism.

## 5. Conclusions

This work demonstrated the comparative effects of several macrolide antimicrobials, along with MON, on the CYP3A-dependent testosterone 6β-hydroxylase activity in bovine liver microsomes. TAO, used as a reference macrolide, was confirmed to be a potent inhibitor of this metabolic reaction, consistent with its previously reported ability to form stable MI complexes with CYP3A isozymes in food-producing animals. In contrast, the absence of inhibition by TIL, TUL, and GAM suggests a limited capacity of these modern macrolides to form MI complexes with CYP3A enzymes in cattle liver. MON alone also inhibited the specific CYP3A-mediated activity under assessment, which may reflect substrate competition for the active site of one or more bovine hepatic CYP3A isoforms.

All macrolides tested significantly inhibited the microsomal metabolism of MON in cattle liver; however, the inhibition produced by TAO was markedly greater than that observed for the other macrolides. Molecular docking analyses indicated that TAO and MON exhibited the highest binding affinities for the active site of CYP3A isozymes, compared with TIL, TUL, and GAM. The concordance between the enzyme patterns observed in vitro and the in silico predictions supports the conclusion that TUL, TIL, and GAM are weaker inhibitors of the CYP3A-mediated MON metabolism. The comparatively modest reduction of MON hepatic metabolism caused by these modern macrolides—commonly administered in cattle feedlots—suggests that the likelihood of a clinically relevant DDI under standard dosing conditions is rather low. Nevertheless, this potential interaction warrants further investigation in vivo, particularly under scenarios involving unintended MON overexposure.

The present study provides original evidence on metabolic interferences among therapeutic drugs used in cattle feedlot systems, with molecular docking analyses reported for the first time for certain macrolides and MON, underscoring their pharmaco-toxicological relevance in cattle production.

## 6. Study Limitations

The use of liver microsomes does not fully reproduce the complexity of hepatic physiology and drug metabolism, and the small number of animals may not capture broader biological variability. In addition, MON inhibition was evaluated at a single macrolide concentration, and the in vitro exposure levels may not accurately reflect actual hepatic concentrations in feedlot cattle. The docking analyses were based on the human CYP3A4 structure in an inhibited conformation, which provided a defined model for assessing the interactions of each macrolide and MON within the CYP3A binding cavity. While this structure shares a high degree of sequence and structural homology with bovine CYP3A, subtle differences in amino acid composition or local conformational flexibility could influence ligand binding. Finally, although the study suggests a low likelihood of clinically relevant DDIs, no in vivo experiments were conducted to determine whether the modest inhibition observed translates into meaningful changes in MON metabolism or toxicity risk under real feedlot conditions. Future research should include in vivo studies involving co-administration of macrolides and MON, particularly under scenarios of unintended ionophore overexposure.

## Figures and Tables

**Table 1:** MS/MS detector parameters for the monensin (MON) quantification method.

Rt (min)	Polarity	Precursor (*m*/*z*)	Quantifier ion (*m*/*z*)	CE (eV)	Qualifier ion (*m*/*z*)	CE (eV)
7.3–7.5	ESI (+)	693.1	461.1	54	479.1	53

Rt: Retention time (range); ESI: Electrospray ionization; CE: Collision energy.

**Table 2:** Comparative O-demethylation of monensin and N-demethylation of four macrolide antimicrobials in cattle liver microsomes.

Enzyme Activity	nmol/min.mg	nmol/min.nmol CYP
MON O-demethylase	0.32 ± 0.06	0.69 ± 0.26
TAO N-demethylase	0.37 ± 0.05	0.80 ± 0.43
TIL N-demethylase	0.37 ± 0.12	0.77 ± 0.31
TUL N-demethylase	0.31 ± 0.08	0.64 ± 0.21
GAM N-demethylase	0.41 ± 0.17	0.89 ± 0.63

Data are the mean (±SD) of four (4) cattle liver microsomal preparations. MON: monensin; TAO: triacetyl-oleandomycin; TIL: tilmicosin; TUL: tulathromycin; GAM: gamithromycin.

**Table 3:** Cytochrome P450 3A4 interacting amino acid residues identified in docking studies with ritonavir (RIT), triacetyloleandomycin (TAO), tilmicosin (TIL), tulathromycin (TUL), gamithromycin (GAM), and monensin (MON). The best binding energy (BBE) corresponding to the most stable conformation identified through docking analysis is presented for each ligand.

	RIT	TAO	MON	TUL	TIL	GAM
**Interacting aminoacid residues**		F57		F57		F57
	R105	R105		R105		R105
	R106	R106	R106	R106	R106	R106
	F108	F108	F108	F108		F108
	S119	S119	S119		S119	S119
	I120	I120		I120		I120
	L210	L210				
	L211	L211			L211	L211
			F213		F213	
			F215			
			T224			
	F304	F304	F304		F304	F304
		A305			A305	
			E308			
	T309	T309	T309	T309	T309	T309
	I369		I369			
	A370		A370			
				R372		
		E374		E374	E374	E374
**BBE (Kcal/mol)**	−10.5	−10.9	−10.6	−10.2	−10.0	−9.9

## Data Availability

The raw data supporting the conclusions of this article will be made available by the authors upon request.

## References

[1] Grandin T. (2016). Evaluation of the welfare of cattle housed in outdoor feedlot pens. Vet. Anim. Sci..

[2] Nebbia C. (2001). Biotransformation enzymes as determinants of xenobiotic toxicity in domestic animals. Vet. J..

[3] Lynch T., Price A. (2007). The effect of cytochrome P450 metabolism on drug response, interactions, and adverse effects. Am. Fam. Physician.

[4] Anadón A., Reeve-Johnson L. (1999). Macrolide antibiotics, drug interactions and microsomal enzymes: Implications for veterinary medicine. Res. Vet. Sci..

[5] Davis J.L., Gookin J.L., Riviere J.E., Papich M.G. (2018). Antiprotozoan drugs. Veterinary Pharmacology and Therapeutics.

[6] Papich M.G., Riviere J.E., Papich M.G. (2018). Chloramphenicol and derivatives, macrolides, lincosamides, and miscellaneous antimicrobials. Veterinary Pharmacology and Therapeutics.

[7] Dubuc J., DuTremblay D., DesCoteaux L., Baril J., Bagg R., Vessie G.H. (2007). Case report: Effects of an unintended high dose of monensin on milk production and milk fat in a dairy herd. Bovine Pract..

[8] Andrade Brito E.S., Gottschalk Andrade T., Sousa de Oliveira C.H., Brianezi Dignani de Moura V.M. (2020). Outbreak of monensin poisoning in cattle due to supplementation error. Ciênc. Rural.

[9] Ensley S.M. (2020). Ionophore use and toxicosis in cattle. Vet. Clin. Food Anim. Pract..

[10] Nebbia C., Ceppa L., Dacasto M., Nachtmann C., Carletti M. (2001). Oxidative monensin metabolism and cytochrome P450 3A content and functions in liver microsomes from horses, pigs, broiler chicks, cattle and rats. J. Vet. Pharmacol. Ther..

[11] Zweers-Zeilmaker W.M., Van Miert A.S., Horbach G.J., Witkamp R.F. (1999). In vitro complex formation and inhibition of hepatic cytochrome P450 activity by different macrolides and tiamulin in goats and cattle. Res. Vet. Sci..

[12] Carletti M., Gusson F., Zaghini A., Dacasto M., Marvasi L., Nebbia C. (2003). In vitro formation of metabolic-intermediate cytochrome P450 complexes in rabbit liver microsomes by tiamulin and various macrolides. Vet. Res..

[13] Basaraba R.J., Oehme F.W., Vorhies M.W., Stokka G.L. (1999). Toxicosis in cattle from concurrent feeding of monensin and dried distiller’s grains contaminated with macrolide antibiotics. J. Vet. Diagn. Investig..

[14] Virkel G., Carletti M., Cantiello M., Della Donna L., Gardini G., Girolami F., Nebbia C. (2010). Characterization of xenobiotic metabolizing enzymes in bovine small intestinal mucosa. J. Vet. Pharmacol. Ther..

[15] Lowry O.H., Rosebrough N.J., Farr A.L., Randall R.J. (1951). Protein measurement with the Folin phenol reagent. J. Biol. Chem..

[16] Matsubara T., Koike M., Touchi A., Tochino Y., Sugeno K. (1976). Quantitative determination of cytochrome P-450 in rat liver homogenate. Anal. Biochem..

[17] Nebbia C., Dacasto M., Rossetto Giaccherino A., Giuliano Albo A., Carletti M. (2003). Comparative expression of liver cytochrome P450-dependent monooxygenases in the horse and in other agricultural and laboratory species. Vet. J..

[18] Werringloer J. (1978). Assay of formaldehyde generated during microsomal oxidation reactions. Methods Enzymol..

[19] Pegolo S., Giantin M., Dacasto M., Montesissa C., Capolongo F. (2010). Testosterone hydroxylation in bovine liver: Enzyme kinetic and inhibition study. Xenobiotica.

[20] Donoho A.L. (1984). Biochemical studies on the fate of monensin in animals and in the environment. J. Anim. Sci..

[21] Giguère S., Huang R., Malinski T.J., Dorr P.M., Tessman R.K., Somerville B.A. (2011). Disposition of gamithromycin in plasma, pulmonary epithelial lining fluid, bronchoalveolar cells, and lung tissue in cattle. Am. J. Vet. Res..

[22] Villarino N., Brown S.A., Martín-Jiménez T. (2014). Understanding the pharmacokinetics of tulathromycin: A pulmonary perspective. J. Vet. Pharmacol. Ther..

[23] Foster D.M., Sylvester H.J., Papich M.G. (2017). Comparison of direct sampling and bronchoalveolar lavage for determining active drug concentrations in the pulmonary epithelial lining fluid of calves injected with enrofloxacin or tilmicosin. J. Vet. Pharmacol. Ther..

[24] The UniProt Consortium (2023). UniProt: The universal protein knowledgebase in 2023. Nucleic Acids Res..

[25] Stothard P. (2000). The Sequence Manipulation Suite: JavaScript programs for analyzing and formatting protein and DNA sequences. Biotechniques.

[26] Meng E.C., Goddard T.D., Pettersen E.F., Couch G.S., Pearson Z.J., Morris J.H., Ferrin T.E. (2023). UCSF ChimeraX: Tools for structure building and analysis. Protein Sci..

[27] Sevrioukova I.F. (2017). High-level production and properties of the cysteine-depleted cytochrome P450 3A4. Biochemistry.

[28] Pettersen E.F., Goddard T.D., Huang C.C., Couch G.S., Greenblatt D.M., Meng E.C., Ferrin T.E. (2004). UCSF Chimera: A visualization system for exploratory research and analysis. J. Comput. Chem..

[29] Trott O., Olson A.J. (2010). AutoDock Vina: Improving the speed and accuracy of docking with a new scoring function, efficient optimization, and multithreading. J. Comput. Chem..

[30] Lodhi S.S., Farmer R., Jaiswal Y.K., Wadhwa G. (2015). In silico structural, virtual screening and docking studies of human cytochrome P450 2A7 protein. Interdiscip. Sci. Comput. Life Sci..

[31] Eberhardt J., Santos-Martins D., Tillack A.F., Forli S. (2021). AutoDock Vina 1.2.0: New docking methods, expanded force field, and Python bindings. J. Chem. Inf. Model..

[32] Adasme M.F., Linnemann K.L., Bolz S.N., Kaiser F., Salentin S., Haupt V.J., Schroeder M. (2021). PLIP 2021: Expanding the protein–ligand interaction profiler to DNA and RNA. Nucleic Acids Res..

[33] Fohner A.E., Sparreboom A., Altman R.B., Klein T.E. (2017). PharmGKB summary: Macrolide antibiotic pathway, pharmacokinetics/pharmacodynamics. Pharmacogenet Genom..

[34] Dacasto M., Eeckhoutte C., Capolongo F., Dupuy J., Carletti M., Calléja C., Nebbia C., Alvinerie M., Galtier P. (2005). Effect of breed and gender on bovine liver cytochrome P450 3A (CYP3A) expression and inter-species comparison with other domestic ruminants. Vet. Res..

[35] Maté M.L., Ballent M., Larsen K., Lifschitz A., Lanusse C., Virkel G. (2015). Gene expression and enzyme function of two cytochrome P450 3A isoenzymes in rat and cattle precision cut liver slices. Xenobiotica.

[36] Cantiello M., Carletti M., Giantin M., Gardini G., Capolongo F., Cascio P., Pauletto M., Girolami F., Dacasto M., Nebbia C. (2022). Induction by Phenobarbital of Phase I and II Xenobiotic-Metabolizing Enzymes in Bovine Liver: An Overall Catalytic and Immunochemical Characterization. Int. J. Mol. Sci..

[37] Nebbia C., Ceppa L., Dacasto M., Carletti M., Nachtmann C. (1999). Oxidative metabolism of monensin in rat liver microsomes and interactions with tiamulin and other chemotherapeutic agents: Evidence for the involvement of cytochrome P-450 3A subfamily. Drug Metab. Dispos..

[38] Kirst H.A., Donoho A.L., Creemer L.C., Wind J.A., Berry D.M., Occolowitz J.L., Paschal J.W. (1994). Identification and synthesis of products isolated during metabolism studies of tilmicosin. J. Agric. Food Chem..

[39] Food and Agriculture Organization of the United Nations (FAO) (2008). Residue Evaluation of Certain Veterinary Drugs: Monographs of the 70th Meeting of the Joint FAO/WHO Expert Committee on Food Additives. FAO JECFA Monographs No. 2. https://www.fao.org/food/food-safety-quality/scientific-advice/jecfa/jecfa-vetdrugs/details/en/c/46/.

[40] European Medicines Agency (EMA) (2015). European Public MRL Assessment Report (EPMAR) for Tulathromycin—Modification of the ADI and MRLs in Bovine and Porcine Species. EMA/CVMP/598235/2013-Rev.2. https://www.ema.europa.eu/en/documents/mrl-report/tulathromycin-modification-microbiological-adi-and-mrls-bovine-and-porcine-species-european-public-maximum-residue-limit-assessment-report-epmar-cvmp_en.pdf.

[41] Huang R.A., Letendre L.T., Banav N., Fischer J., Somerville B. (2010). Pharmacokinetics of gamithromycin in cattle with comparison of plasma and lung tissue concentrations and plasma antibacterial activity. J. Vet. Pharmacol. Ther..

[42] Ceppa L., Dacasto M., Carletti M., Montesissa C., Nebbia C. (1997). ‘In vitro’ interactions of monensin with hepatic xenobiotic metabolizing enzymes. Pharmacol. Res..

[43] Szucs G., Tamási V., Laczay P., Monostory K. (2004). Biochemical background of toxic interaction between tiamulin and monensin. Chem. Biol. Interact..

[44] Puschner B., Bautista A.C., McKemie D.S., Gallego S.M., Woods L.W., Moore C.E., Knych H.K. (2016). Serum, milk, and tissue monensin concentrations in cattle with adequate and potentially toxic dietary levels of monensin: Pharmacokinetics and diagnostic interpretation. J. Vet. Pharmacol. Ther..

[45] Giantin M., Rahnasto-Rilla M., Tolosi R., Lucatello L., Pauletto M., Guerra G., Pezzato F., Lopparelli R.M., Merlanti R., Carnier P. (2019). Functional impact of cytochrome P450 3A (CYP3A) missense variants in cattle. Sci. Rep..

[46] Iori S., Lahtela-Kakkonen M., D’Onofrio C., Maietti F., Mucignat G., Bardhi A., Barbarossa A., Zaghini A., Pauletto M., Dacasto M. (2024). New insights into aflatoxin B1 mechanistic toxicology in cattle liver: An integrated approach using molecular docking and biological evaluation in CYP1A1 and CYP3A74 knockout BFH12 cell lines. Arch. Toxicol..

[47] Loos N.H.C., Beijnen J.H., Schinkel A.H. (2022). The Mechanism-Based Inactivation of CYP3A4 by Ritonavir: What Mechanism?. Int. J. Mol. Sci..

[48] Tanaka T., Okuda T., Yamamoto Y. (2004). Characterization of the CYP3A4 active site by homology modeling. Chem. Pharm. Bull..

[49] Pedroni L., Gehring R., Dorne J.C.M., Girolami F., Nebbia C., Dellafiora L. (2026). In Silico molecular insights into CYP3A-mediated monensin detoxification across species. Toxicology.

[50] American Veterinary Medical Association (2007). AVMA Guidelines on Euthanasia [Internet].

